# DRPChain: A new blockchain-based trusted DRM scheme for image content protection

**DOI:** 10.1371/journal.pone.0309743

**Published:** 2024-09-19

**Authors:** Jian Yun, Xinyu Liu, Yusheng Lu, Jingdan Guan, Xinyang Liu

**Affiliations:** College of Computer Science and Engineering, Dalian Minzu University, Dalian, China; Military Institute of Science and Technology, BANGLADESH

## Abstract

The unauthorized replication and distribution of digital images pose significant challenges to copyright protection. While existing solutions incorporate blockchain-based techniques such as perceptual hashing and digital watermarking, they lack large-scale experimental validation and a dedicated blockchain consensus protocol for image copyright management. This paper introduces DRPChain, a novel digital image copyright management system that addresses these issues. DRPChain employs an efficient cropping-resistant robust image hashing algorithm to defend against 14 common image attacks, demonstrating an 85% success rate in watermark extraction, 10% higher than the original scheme. Moreover, the paper designs the K-Raft consensus algorithm tailored for image copyright protection. Comparative experiments with Raft and benchmarking against PoW and PBFT algorithms show that K-Raft reduces block error rates by 2%, improves efficiency by 300ms compared to Raft, and exhibits superior efficiency,decentralization, and throughput compared to PoW and PBFT. These advantages make K-Raft more suitable for digital image copyright protection. This research contributes valuable insights into using blockchain technology for digital copyright protection, providing a solid foundation for future exploration.

## Introduction

The ease of replicating digital images has facilitated the unauthorized appropriation of copyrights, causing significant losses to image creators. The challenges in identifying digital image infringement and the lengthy legal redress process inadvertently contribute to the proliferation of piracy. To effectively counter this trend, establishing a robust Digital Rights Management (DRM) system for image data is pivotal for regulating the legitimate use of visual content. Such a system should encompass content registration, verification of authenticity and legality, and decentralized functionalities.

The first issue that needs to be clarified is the manner in which content is registered. Typically, digital watermarking technology embeds hidden information such as copyright statements within digital works, thereby registering the digital image. These watermarks are designed for copyright protection and must remain detectable and extractable even after various operations or attacks to ensure the recognizability of copyright information. This approach has demonstrated practicality and effectiveness in real-world applications.

Additionally, verifying the authenticity and legality of content is the core function of a copyright protection system. Traditional encryption hash algorithms like MD5 and SHA256 produce significantly different hash values due to the modification of digital image content structure and the addition, filtering, rotation, and compression of digital watermarks. However, these differences do not result in perceptual differences in the human visual system and, therefore, cannot accurately assess image similarity. In contrast, perceptual hashing algorithms can produce consistent or similar computational results while maintaining the overall structure of the image, aligning with human perception. Such algorithms provide a reliable foundation for determining copyright infringement by measuring image similarity.

High content availability is crucial for a DRM system, so an ideal model should be built on a distributed peer-to-peer (P2P) network infrastructure to support a distributed ledger technology (DLT) environment. With its reliance on a global P2P network rather than a centralized trusted authority, blockchain technology is an ideal choice for recording transaction history and addressing content privacy and security concerns.

To achieve the main functionalities of the proposed DRM, this paper presents an innovative digital image copyright management system with the following contributions:

Firstly, the system utilizes blockchain technology to encapsulate original digital images and their related metadata into blockchain transactions, designing a three-stage digital image copyright registration process that includes transaction encapsulation, candidate block generation, and candidate block validation.

Secondly, considering the uniqueness of digital images, the system implements an anti-cropping originality judgment and retrieval system based on high robustness perceptual hashing values.

Lastly, to meet the demand for decentralized copyright protection, the system introduces an innovative ReSolver election mechanism to establish proof of content (PoC).This mechanism selects leader nodes by measuring a digital image’s perceptual hashing Hamming distance. Additionally, the system designs a multi-leader collaboration mechanism to address the leader soft switch issue in the Raft consensus algorithm. This mechanism allows multiple leader nodes to temporarily coexist in the system, thereby enhancing system efficiency and reducing the error rate of block generation.

The abbreviations and meanings of related terms are listed in the Table 1.

**Table 1:** Acronym description.

Acronym	Explanation
DRM	Digital Rights Management
P2P	Peer-to-Peer
DLT	Distributed Ledger Technology
REM	ReSolver Election Mechanism
PoW	Proof of Work
PoS	Proof of Stake
DPoS	Delegated Proof of Stake
PBFT	Practical Byzantine Fault Tolerance
PoC	Proof of Content
MLCM	Multi-Leader Collaboration Mechanism
CTP	Confirmation Tensor Pool

## Related works

As a decentralized and immutable distributed ledger, blockchain technology comprises multiple blocks interconnected through encryption technology. It facilitates data transmission via a peer-to-peer network and relies on consensus mechanisms to maintain data consistency and security across the network [1]. Depending on the degree of decentralization, blockchains can be categorized into public chains [2, 3], private chains, and consortium chains [4], each suitable for specific application scenarios.

Consensus algorithms are the core of blockchain technology, including Proof of Work (PoW) [2] and Proof of Stake (PoS) [5], each with its unique advantages and limitations. For example, PoW provides high security but consumes vast energy. At the same time, PoS and its variant DPoS [6] are more efficient and consume less energy, although they may introduce centralization risks. Blockchain technology has shown extensive application potential in areas such as supply chain management, intellectual property rights, electronic voting, finance [7], the Internet of Things [8–11], and medical data protection [12–16]. These applications provide innovative data traceability, security, and privacy protection solutions [17].

In digital rights management, blockchain technology can store image content metadata and data in an off-chain trusted storage system for complex computations. As blockchain technology evolves, many scholars have explored its use in solving digital copyright protection issues. Studies such as Tresise et al. [18] and Savelyev [19] have analyzed the interaction between copyright law and blockchain technology, discussing the application of Blockchain in copyright protection.Ma et al. [20] proposed a blockchain-based digital rights management (DRMChain) solution, storing original and protected digital content through the BAI interface and introducing an efficient and secure multi-signature method for authentication, privacy protection, and conditional tracking.Guo et al. [21] introduced a blockchain digital rights management system for multimedia resource sharing and management in online education.Jing et al. [22] proposed a code originality verification model based on Abstract Syntax Trees (AST), comparing uploaded code with other original code to determine originality and storing original code copyright information on the blockchain network.Natgunanathan et al. [23] developed a multi-level watermarking mechanism to protect multimedia data in Multimedia Distribution Networks (MDNs) and enforced correct operations through smart contracts.

Zero-watermarking technology [24–26] is a watermarking technique employed in the field of digital rights management that diverges from traditional digital watermarking practices. Unlike conventional methods, zero-watermarking does not embed information directly into the original data but instead constructs an external watermark model with specific associations to the original data. During copyright verification, the watermark model of the current data is compared with the original model to ascertain data integrity and copyright ownership. However, traditional watermarking methods depend on trusted third-party arbitration, which may introduce security risks and cost concerns. Moreover, the watermark embedding algorithms can damage image data, hindering the traceability of image ownership. Consequently, it is imperative to integrate blockchain technology with distributed storage systems and an image copyright protection framework. This framework eliminates the need for a trusted third party, enabling traceability, integrity, and automated verification of image copyrights with reduced costs and enhanced scalability. Meng et al. [27] introduce a digital image copyright management system based on perceptual hash functions, blockchain, and IPFS. However, more detailed and formal design explanations are needed, and typical attack test experiments have yet to be conducted to assess the practical feasibility of the proposed solution.Garba et al. [28] present a distributed media trading framework based on digital watermarking and an expandable blockchain model. The framework achieves blockchain scalability through an overlay network and implements digital copyright protection using a Discrete Cosine Transform (DCT) digital watermarking scheme [29]. Kumar et al. [30] outline a distributed detection system based on IPFS and blockchain technology for safeguarding the copyright of industrial image and video data. The system utilizes perceptual hash technology to detect copyright infringements of multimedia works, storing multimedia files on IPFS and their perceptual hash values on the blockchain network. The system detects copyright infringement when a newly uploaded multimedia file’s perceptual hash value exceeds a 50% similarity threshold with those on the chain. The literature [31] introduces a secure image copyright protection system based on blockchain.This system combines zero-watermarking algorithms, the Ethereum blockchain, and the distributed storage system IPFS. Image owners can generate feature maps and watermark images using zero-watermarking algorithms and store them in IPFS. Authorized users can query image owner information on the blockchain and verify image copyright using zero-watermarking algorithms. The literature [32] proposes an image copyright protection method that integrates zero-watermarking with blockchain technology. The method extracts unique watermark information from images using zero-watermarking technology without modifying the image content. The paper constructs a copyright protection framework based on the Ethereum blockchain to overcome the vulnerabilities of traditional zero-watermarking techniques that rely on trusted third-party storage. Combined with IPFS distributed storage, it addresses the efficiency issue of blockchain storage for large files and implements copyright information registration, image query, and transaction functions through smart contracts. The literature [33] presents a digital rights management scheme based on redactable blockchain and perceptual hash. The scheme uses perceptual hash to detect similarity and determine whether digital content is original. Additionally, it proposes an incentive scheme based on a redactable blockchain to ensure that illegal, sensitive, and plagiarized digital content can be deleted from the blockchain in a timely manner. The literature [34] proposes a vector map copyright protection framework that integrates zero-watermarking technology with blockchain. The framework constructs a zero-watermark sequence by extracting corner features of line and surface elements of vector maps, achieving strong resistance to attacks. Leveraging blockchain’s decentralized characteristics ensures copyright information’s credibility and traceability. The literature [28] introduces a digital rights management system based on a scalable blockchain. The system enhances blockchain scalability and throughput through overlay networks and practical Byzantine Fault Tolerance consensus algorithms. It enhances copyright declarations and content security by embedding digital watermarks and lightweight encryption algorithms. Users obtain session keys after payment to access cloud-stored content, with transaction records stored on the blockchain. The blockchain tracks copyright transfer and content modifications, and smart contracts enable automatic payment and incentives.

In summary, most research in this area has combined watermarking and blockchain technologies to achieve traceability and immutability of multimedia data. Most of these studies have adopted existing public blockchain platforms and their consensus algorithms, such as PoW and PoS. However, there needs to be more blockchain consensus algorithms tailored to specific copyright protection scenarios. This study fills this gap with a comparison of the proposed scheme with existing research schemes presented in Table 2.

**Table 2:** Scheme comparison.

Related Work	Year	Blockchain Platform	Consensus Algoritim
Wang et al. [31]	2020	Ethereum	PoW
Kumar et al. [30]	2021	Custom Blockchain Platform	PoW
Ren et al. [34]	2021	Ant Blockchain Open Alliance	Not explicitly mentioned
Garba et al. [28]	2021	permissioned blockchain	PBFT
Chen et al. [32]	2022	Ethereum	Not explicitly mentioned
Yi et al. [33]	2023	redactable blockchain	Not explicitly mentioned
This Scheme		DRPchain	K-raft

## Materials and methods

This section concentrates on two pivotal issues in digital image copyright protection: watermark embedding and similarity search. We begin by delineating the Discrete Cosine Transform (DCT), a fundamental technique for transitioning signals from the spatial domain to the frequency domain, which is widely utilized in image processing, including JPEG compression. Subsequently, we introduce the Perceptual Hashing Algorithm (PHA), which enables rapid comparison of image similarity by extracting features and generating fingerprints. We then delve into the application of image watermarking for copyright protection, proposing an embedding scheme that utilizes a perceptual hash-based QR-code as the watermark, along with an algorithm for its generation. Lastly, we discuss the Hamming distance, a metric for calculating the disparity between binary vectors, which finds significant application in image similarity search, as well as in the computation of distances between blockchain transactions and nodes.

### The Discrete Cosine Transform

The two-dimensional continuous image f(x,y) is divided into N rows and M columns. The intersection of a row and a column is called a pixel. The value assigned to the integer coordinates [m,n], where {m = 0,1,2,…, M-1} and {n = 0,1,2,…, N-1}, is f[m,n]. In practice, in most cases, we can consider f(x,y), which we might think of as the actual signal impinging on the surface of a two-dimensional sensor, to be a function of many variables, including depth (z), color (l), and time (t). Unless otherwise stated, we will consider the case of a two-dimensional, monochrome, static image in this section.

The Discrete Cosine Transform (DCT) is a method for converting signals from the spatial domain to the frequency domain. DCT is an orthogonal transform with a transformation kernel being the cosine function. By decomposing a signal into a linear combination of sine and cosine functions with frequencies that correspond to differences between adjacent values in the original signal, DCT can be used to analyze the frequency components of a signal. It is widely used in image processing, such as in JPEG compression. There are two directions of DCT:

DCT(shown in Eq 1) which transforms from the spatial domain to the frequency domain, and IDCT(shown in Eq 2), which transforms from the frequency domain back to the spatial domain.

$$\begin{array}{c} \begin{array}{cc} & F\left(\mu ,\nu \right)=c\left(\mu \right)c\left(\nu \right)\sum_{x=0}^{M-1}\sum_{y=0}^{N-1}f\left(x,y\right)\;\text{cos}\;\frac{\pi (2x+1)}{2M}\text{cos}\frac{\pi (2y+1)}{2N}\\ & \mu =0,1,\ldots ,\text{M}-1;\nu =0,1,\ldots ,\text{N}-1 \end{array} \end{array}$$ (1)

$$\begin{array}{c} \begin{array}{cc} & \text{f}(\text{x},\text{y})=\sum_{x=0}^{M-1}\sum_{y=0}^{N-1}c\left(\mu \right)c\left(\nu \right)F\left(\mu ,\nu \right)cos\frac{\pi (2x+1)\mu }{2M}cos\frac{\pi (2y+1)\nu }{2N}\\ & \text{x}=0,1,\ldots ,\text{M}-1;\text{y}=0,1,\ldots ,\text{N}-1 \end{array} \end{array}$$ (2)

$$\begin{array}{c} c\left(\mu \right)=\{\begin{array}{cc} \sqrt{\frac{1}{M}} & \mu =0\\ \sqrt{\frac{2}{M}} & \mu =1,2,\ldots ,M-1 \end{array}c\left(\nu \right)=\{\begin{array}{cc} \sqrt{\frac{1}{M}} & \nu =0\\ \sqrt{\frac{2}{N}} & \nu =1,2,\ldots N-1 \end{array} \end{array}$$ (3)

### Perceptual hash algorithm

**Fig 1:**
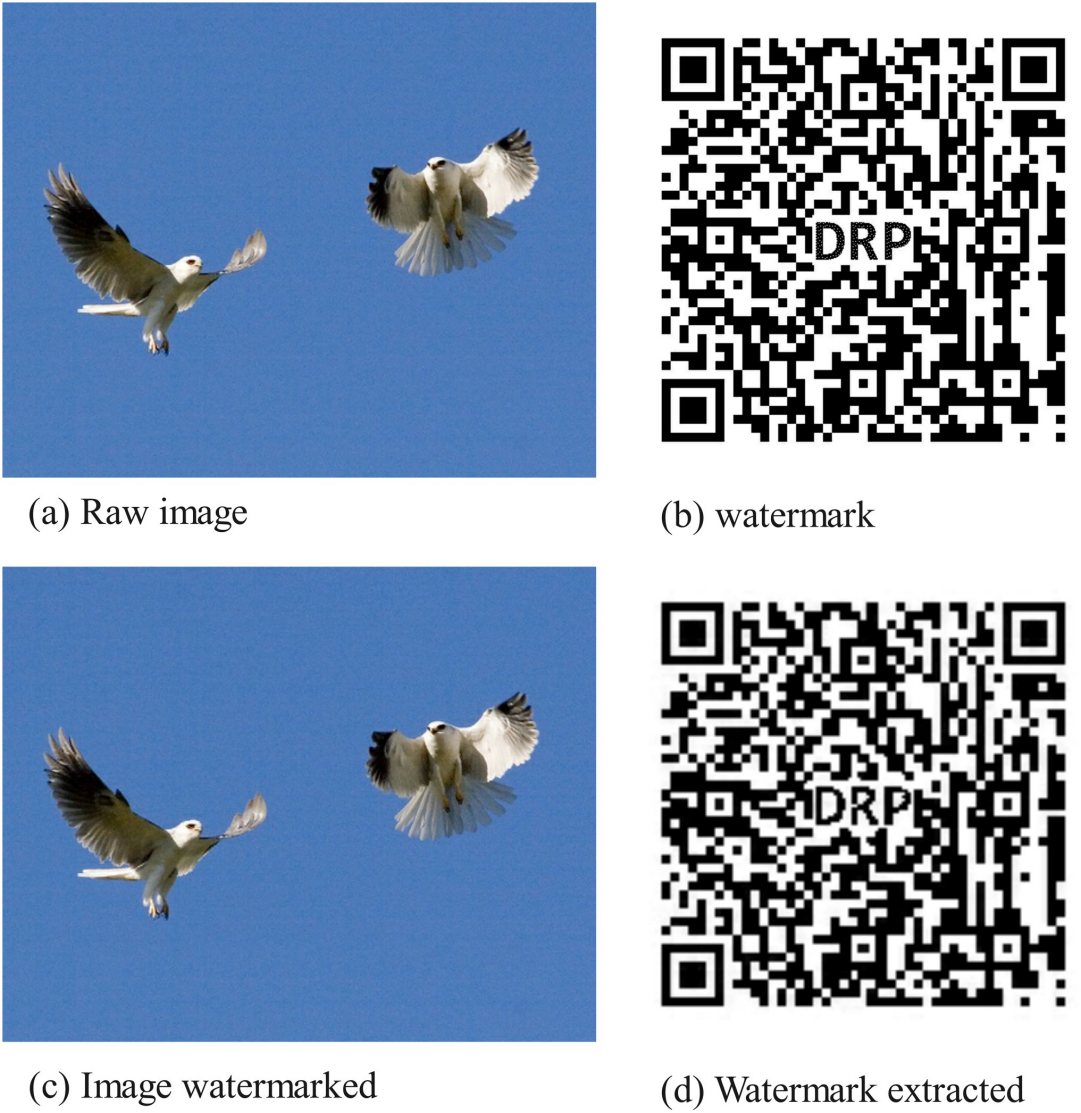
The process of adding and parsing watermarks in images.

Traditional encryption hash algorithms such as MD5 and SHA256 are unsuitable for generating hash values from digital images due to tampering attacks on the content structure and routine operations such as adding watermarks, filtering, rotation, and compression. These operations do not cause perceptual differences that human sensory systems can detect; therefore, they remain the same as the original image. However, the data structure of digital image files has changed due to computers, resulting in entirely different hash values calculated by traditional hash functions. As shown in Fig 1 and Table 3, the original image, the watermark, the watermarked image, and the extracted watermark are respectively presented. The perceptual hashes of the original and watermarked images are the same but different from traditional cryptographic hashes. The situation of the watermark and the extracted watermark is also similar.

**Table 3:** Perceptual hashing and traditional cryptographic hashing of different images.

Image Type	Perceptual Hash	Cryptographic Hash
Raw image	8d25f05a0fa5f0da	4e2bac6425ba865db22b8486337604b 9a7734670ec617afe8026401bf97ad0bd
Watermark	ff92916c81cae463	426e44179078c6a68029dc519c9aa568 22950181567ec4fe18252070e557f674
Image watermarked	8d25f05a0fa5f0da	fc83c778fd183014c7cfc4f837a7f827c 10f120ff58e46d3663dad7530586326
Watermark extracted	ff92916c81cae463	de07f4961dffac92887d393fc9d7dbd47 7a0d699e24dfaa76bdccb6255f5f9a9

A perceptual hash algorithm (PHA) can be used for image similarity search by extracting features from images and generating a set of fingerprints, which are then compared to determine image similarity. The closer the results are, the more similar the images are. PHA includes Mean Hash Algorithm (AHA), Perceptual Hash Algorithm (PHA), and Different Hash Algorithm (DHA). This paper adopts PHA for image similarity search [35]. Efficient cropping-resistant robust image hashing is a highly robust perceptual hash algorithm against image cropping attacks. It can use the above perceptual hash functions to calculate and adopt an image segmentation mechanism to divide the image into multiple subregions containing large objects. A hash is generated for each subregion, ultimately forming a hash database of the entire image. Since the algorithm generates hashes on different objects in the image, the final matching is based on the similarity between objects of the image, making it effective in resisting cropping attacks. We use this algorithm to resist cropping attacks that are not resistant to the original scheme.

**Step 1:** convert the original 3 channels image to grayscale:
$$\begin{array}{c} g\left[x,y\right]=\frac{1}{3}\sum_{i=1}^{3}a{\left[x,y\right]}_{z} \end{array}$$ (4)
**Step 2:** calculate the DCT by transforming the grayscale image from the spatial domain to the frequency domain:
$$\begin{array}{c} G[\mu ,\nu ]=\text{DCT}(g[x,y]) \end{array}$$ (5)
**Step 3:** Only the upper left *k* × *k* of the transformed image is retained. These represent the lowest frequencies in the image and capture the basic features of the image. calculate the mean DCT value:
$$\begin{array}{c} RG.flattern=G[:k,:k].flattern \end{array}$$ (6)
$$\begin{array}{c} mRG=\frac{1}{k^{2}}\sum_{i=1}^{k^{2}}RG.flattern\left[i\right] \end{array}$$ (7)
**Step 4:** construct the hash for each of the *k*^2^ DCT values, set either a 0 or a 1 bit based on whether it is above or below the mean value. The result does not tell us the actual low frequencies; it only gives us a rough proportion of the frequencies relative to the mean value. As long as the overall structure of the image remains unchanged, the result will not change; this can withstand gamma and color histogram adjustments without problems. The *k*^2^ bits are set into a *k*^2^-bit integer. The PHA values can be compared using the same Hamming distance algorithm.

Efficient Cropping-Resistant Robust Image Hashing [36] assumes that even after cropping, at least several enormous objects of an image will be left. The method first segments the image based on the watershed segmentation. A set of 256-bit hashes identifies each segment of the image, and the distance is calculated by voting to obtain the smallest segment hash distance between two images.

### Image watermarking

Because QR-codes can carry certain information and have good robustness, this paper continues the scheme by Meng et al. [27] of using QR-codes to generate watermark images. QR-codes can still be successfully detected and read when data is lost from 7%—30%. The difference is that this paper uses a scheme that generates QR-codes with block information called *TokenSignature* embedded in the original image.When the block is successfully uploaded to the blockchain, the embedded watermarked image is uploaded to IPFS for storage. A process of watermarked image generation is shown in algorithm 1.

### Hamming distance

The Hamming distance(*HD*) is used to calculate the distance between transactions and nodes, as well as between digital images. In this system, the Hamming distance describes the number of differing bits at corresponding positions in two binary vectors of equal length. Given two vectors of length n, $\vec{a}=\left[a_{0},a_{1},\ldots a_{n-1}\right],\vec{b}=\left[b_{0},b_{1},\ldots ,b_{n-1}\right]$, the Hamming distance between them is defined as:

$$\begin{array}{c} HD=\sum_{i=0}^{n-1}\left(a_{i}⊕b_{i}\right) \end{array}$$ (8)

The Hamming distance between a given vector of length n $\vec{a}$ and a matrix **B** containing m vectors of length n $\vec{b}$ is defined as:

$$\begin{array}{c} HDs=\{d|HD\left(\vec{a},\vec{b}\right),\;\forall \;\vec{b}\;\in \text{B}\} \end{array}$$ (9)

**Algorithm 1** Generation of watermarked image

**Input**: *Image as a*[*x*, *y*], *CopyRightData as CRD*, *MetaData as MD*

**Output**: *WatermarkedImage as aw*[*x*, *y*]

  // Getting image cryptographic hash.

1: *ICH*=hash.sha256(*a*[*x*, *y*])

2: *IPH*=calculcate perceptual hash of *a*[*x*, *y*].

  // Getting TokenSignature.

3: *TS* = list of *ICH*, *IPH*, *CRD*, *MD*.

  // Get watermark from tokenSignature.

4: *w*[*x*′, *y*′] = QrCode(*TS*)

5: *W*[*μ*′, *ν*′] = DCT(*w*[*x*′, *y*′])

  // Combinating image DCT blocks with watermark pixels.

6: **for** *i*, *pixel* in *W*[*μ*′, *ν*′] **do**

7:  *AW*[*μ*, *ν*].append(Comb(DCT(*A*[*μ*, *ν*].*block*[*i*]), *pixel*))

8: **end for**

  // Return the watermarked image.

9: *aw*[*x*, *y*] = IDCT(*AW*[*μ*, *ν*])

## Solution design

For ease of understanding, all variables and their meanings involved in this study are listed in the Table 4.

**Table 4:** Variables and meanings.

Variable	Meaning
*MD*	Meta data
*CP*	Content provider
*TX*	Transactions
*RawImage*	Raw image data
*RawCryptoHash*	Cryptographic hashes of image data
*CopyRightData*	Copyright meta data of a raw image
*RawSize*	Image size information
*CB*	Candidate block
*WmdPhash*	The perceptual hash value of the watermarked image
*WmdCryptoHash*	Cryptographic hash of the watermarked image
*TokenSignature*	Token signature of the original image data
*Provider*	Information of the content provider node
*Proof*	Proof of hamming distance calculating

DRPChain employs an enhanced K-Raft consensus algorithm, which introduces a decentralized node selection mechanism based on the Kademlia protocol. This mechanism selects the nearest node as a leader through file hash values, thereby streamlining the complex log-index-based election process inherent in the traditional Raft algorithm. Furthermore, K-Raft allows for the concurrent existence of multiple leaders, addressing block ordering through a multi-leader collaboration scheme that nhances system efficiency and fault tolerance. The consensus process is modularized into three phases, facilitating a structured and phased approach. For block broadcasting and storage, K-Raft mandates that blocks be broadcast only after consent from at least two-thirds of the nodes, with all nodes subsequently updating the confirmation tensor pool to ensure consensus. To prevent the propagation of erroneous blocks, K-Raft incorporates various verification mechanisms, including validation of leader legitimacy, perceptual hash consistency, proof of originality, and watermark extraction. These refinements render K-Raft a more efficient and resilient consensus mechanism, particularly suited for the copyright protection of image data within the DRPChain ecosystem. A detailed exposition of these features and their implications follows in this section.

### Goals

The Digital Right Management System(DRMS) leverages blockchain technology to facilitate the registration of original content, authenticate authenticity and legality, and promote decentralization. Capitalizing on blockchain’s inherent traceability and immutability, the system employs consensus mechanisms to ensure the dissemination and tracking of original content. It employs similarity-matching techniques to safeguard originality, mitigate copyright disputes, and defend against attacks such as registering similar images. Users are empowered to request similarity reports to uphold their rights. The DRMS incorporates an efficient blockchain consensus algorithm, reducing computational overhead. Subsequent sections will elaborate on these objectives and their implementation within the system.

- **Original content registering**: Relying on the traceability, tamper-proof, and decentralized characteristics of blockchain, combining the digital image copyright registration with the blockchain consensus process can achieve the distribution and tracking of original digital content.
- **Determining the authenticity and legality of content**: Implementation of similarity matching based on semantic features of digital images. When digital images are attacked by common attacks such as rotation, noise, and cropping [35], the scheme can still accurately confirm the originality of the digital images. In the registration stage, it can prevent digital images with high similarity to the existing digital images in the system from being registered, avoiding copyright disputes. During the confirmation of rights, users can query the system to obtain a similarity report between similar images and the registered content of the copyright owner, thereby protecting their legitimate rights and interests.
- **Decentralization**: Implementing a more practical blockchain consensus algorithm, which enables a more decentralized, random, and less computationally expensive selection of block-producing nodes, holds significant importance in digital copyright protection.

### Concepts

To fulfill the objectives above, this paper delineates the constructs of participants, transactions, proposed blocks, and blocks in conjunction with the Confirmation Tensor Pool (CTP). These conceptual definitions lay the foundation for the primary functionalities of a digital image copyright management system.

**Fig 2:**
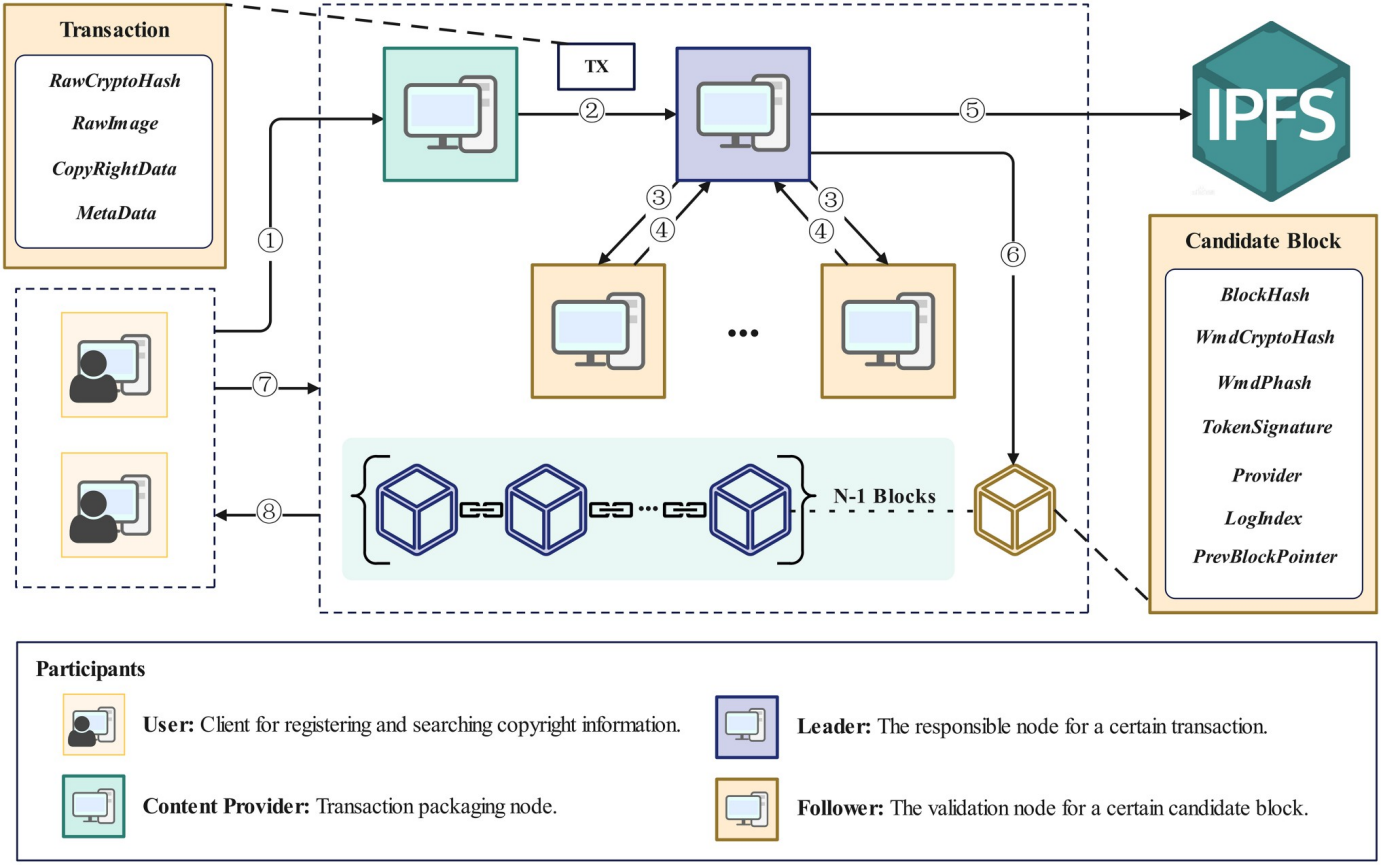
System architecture.

- **Participants**: All the blockchain nodes participating in the consensus process are referred to as participants in this paper, including content providers(*CP*), followers, and leaders. Fig 2 shows all participants of the system.
- **Transactions**: A transaction includes raw image data (*RawImage*), cryptographic hashes of image data (*RawCryptoHash*), copyright information data (*CopyRightData*), including author, title, brief introduction, and metadata including timestamp, image size information (*RawSize*). A content provider will call the CALL_BUILD_BLOCK (*transaction*) to inform the selected leader node to build a candidate block.
- **Candidate Blocks**: Candidate blocks are intermediate state generated by leader nodes, including the perceptual hash value of the watermarked image (*WmdPhash*), cryptographic hash of the watermarked image (*WmdCryptoHash*), token signature of the original image data (*TokenSignature*), information of the content provider node (*Provider*), proof of hamming distance calculating (*Proof*) and local log length of the leader node (*LogIndex*).
- **Blocks and confirmation tensor pool(CTP)**: For digital copyright protection, it is suitable to save the metadata of multimedia data content on the blockchain but store the data itself in an off-chain trusted storage space for complex computation. After a candidate block is voted successfully, the perceptual hash of the watermarked image in the candidate block and the IPFS address are synced to all blockchain nodes as a log. The perceptual hash in binary representation is added to each node’s confirmation tensor pool for large-scale retrieval of existing records (infringement and rights protection detection). The IPFS address ensures the availability of the original image data. The synchronization of logs is based on the Raft consensus algorithm.

**Algorithm 2** Build a candidate block.

**Input**: *A transaction as TX*.

**Output**: *A candidate block as CB*.

  // The node will be a leader.

1: *Local*.*State*=“Receive Tx Phase”

2: *wmd*[*x*, *y*] = *Generate Watermarked Image from TX*.

  // Getting watermarked cryptographic hash.

3: *WCH*=hash.sha256(*wmd*[*x*, *y*])

  // Calculating perceptual hash of watermarked image.

4: *WPH*= calculate perceptual hash of *wmd*[*x*, *y*]

5: **if** *Local*.*CTP* is not empty **then**

6:  *min*_*loc*, *min*_*hd*=calculate hamming distance between *WPH* and *Leader*.*CTP*

  // Generating a proof of the caculation.

7:  *PROOF*=hash.sha256(*min*_*loc*:*Local*.*CTP*[0])

8: **end if**

  // log index is the count of confirmation tensor pool item.

9: *LOG*_*INDEX*=Len(*Local*.*CTP*)

  // *TokenSignature* of this image as TS,*PROVIDER* is the content provider of the image.

10: *CB*=list of *WCH*, *WPH*, *TS*, *aw*[*x*, *y*], *PROVIDER*, *PROOF*, *LOG*_*INDEX*

11: *Local*.*State* = “Broadcast Block Phase”

12: CALL_VALIDATE_BLOCK(*CB*)

The overall operation flow of the system is shown in Fig 2, and the details are as follows:

- **Step 1**: The user uploads image data and copyright information to a node to protect image copyright. The node becomes a content provider. The content provider generates a cryptographic hash value based on the image data and copyright information(shown in Eq 10) and selects a node as leader responsible for uploading the image data to the blockchain.
  $$\begin{array}{c} ICH=\text{hash.sha256}(a[x,y]:CopyRightData) \end{array}$$ (10)
  The leader is the first one returned from the *β*(set *β*=3) nearest nodes calculated Eq 11 based on the Hamming distance.
  $$\begin{array}{c} Leader=\text{Nearest}\;\text{HDs}(ICH,CP.routing\_table,\beta ) \end{array}$$ (11)
- **Step 2**: After choosing a leader, the content provider packages the image data and copyright information into a transaction and sends it to a node. The node becomes the leader node responsible for the image data.
- **Step 3**: The leader node generates and broadcasts a candidate block to all nodes. Other nodes validate the legitimacy of the block, including the leader’s legitimacy, perceptual hash consistency, originality check, and watermark verification. The leader’s legitimacy is verified by checking if the leader node is the closest *β* node to this transaction and if the leader node’s log length is greater than or equal to the local log length of the follower for validation:.
  $$\begin{array}{c} CB.LOG\_INDEX\geq \text{Len}(Local.CTP) \end{array}$$ (12)
  Perceptual hash consistency verifies if the perceptual hash computed locally by the follower is consistent with the perceptual hash in the candidate block:
  $$\begin{array}{c} CB.WPH≟\text{PHA}\left(CB.aw\left[x,y\right]\right) \end{array}$$ (13)
  After that, the originality proof performs the function HDs() on the candidate block’s perceptual hash and the follower’s confirmation tensor pool for validation. If the distance between all hash entries in the candidate block’s perceptual hash and the confirmation tensor pool of the follower is greater than *k*^2^ × 0.85 (such as using a 64-bit perceptual hash, hamming distance greater than 10 which means the similarity between two images is less than 85%), it passes the originality check(same process at line 5–7 of algorithm 2).
  $$\begin{array}{c} \text{bitwise\_XOR}(CB.WPH,Local.CTP^{T}) \end{array}$$ (14)
  Finally, a watermark extraction check is performed. If complete watermark information can be extracted, it proves that the added watermarked image generated can meet the traceability standard.
- **Step 4**: After four verification steps above, the follower votes for the candidate block. The *VotingResult* includes whether to agree to produce a block, the reason for opposing the block (*Reason*), *LOG*_*INDEX*, *PROOF*, and the status of the followers of the candidate block(*State*), which includes four statuses: Follower, Receive Tx Phase, Broadcast Block Phase, and Accept Block Phase, which is provided for the Leader responsible for the candidate block to refer to for block sorting.
  $$\begin{array}{c} VoteResult=[⊤/⊥,Reason,PROOF,LOG\_INDEX,FollowerState] \end{array}$$ (15)
- **Steps 5 and 6**: The leader prepares to store the watermarked image in IPFS after receiving $\frac{2}{3}$ nodes’ favor votes. If The watermarked image is stored in IPFS, the block will broadcast to all nodes in the network. All nodes update their local confirmation tensor pool. Detail is shown in algorithm 3.
- **Steps 7 and 8**: The user can interact with the system to verify the ownership information of the image data and detect infringement.

**Algorithm 3** Validate the voting result and generate block.

**Input**: *VotingResult from followers as VR*.

**Output**: *BuildingResult* as *BR*.

1: *Leader* collects all *VotingResults* from followers.

2: Check all *VR*.*PROOF* and votes grouped by *VR*.*PROOF*.

3: **if** Count(*VR*.⊤) ≥ Count(*cluster*) × $\frac{2}{3}$ **then**

4:  *Local*.*State* = “Accept Block Phase”

  // Leader sorts block by checking states of follower voted.

5:  Check all *VR*.*State* and sort block.

6:  *Loc*_*IPFS*=IPFS_STORE(*Block*)

7:  *Local*.*CTP*.append(*Block*.*WPH*)

8: **else**

9:  **return** *BR*(⊥,*BuildFailureReason*) to the content provider.

10: **end if**

11: CALL_STORE(*Local*.*CTP*[*VR*.*LOG*_*INDEX*:])

12: *Local*.*State* = “Follower”

13: **return** *BR*(⊤,*Loc*_*IPFS*) to the content provider.

### Resolver Election Mechanism

Kademlia defines the structure of the network and how node queries exchange information. Communication nodes form a virtual network, and nodes identify themselves and locate values through their node IDs. The node IDs directly correspond to file hash values, representing the association between files and their stored value at the corresponding node. When searching for the hash of a stored file, Kademlia algorithm recursively searches for keys closer to the target hash step by step until a node directly returns the searched value or no more nodes can be found with keys closer to the target hash. Kademlia only accesses *O(log(n))* nodes. Additionally, this non-centralized network structure significantly enhances resistance to denial-of-service attacks. When a batch of nodes in the network suffers from flood attacks, the network can be reorganized by bypassing the attacked nodes, and network availability can be restored.

**Fig 3:**
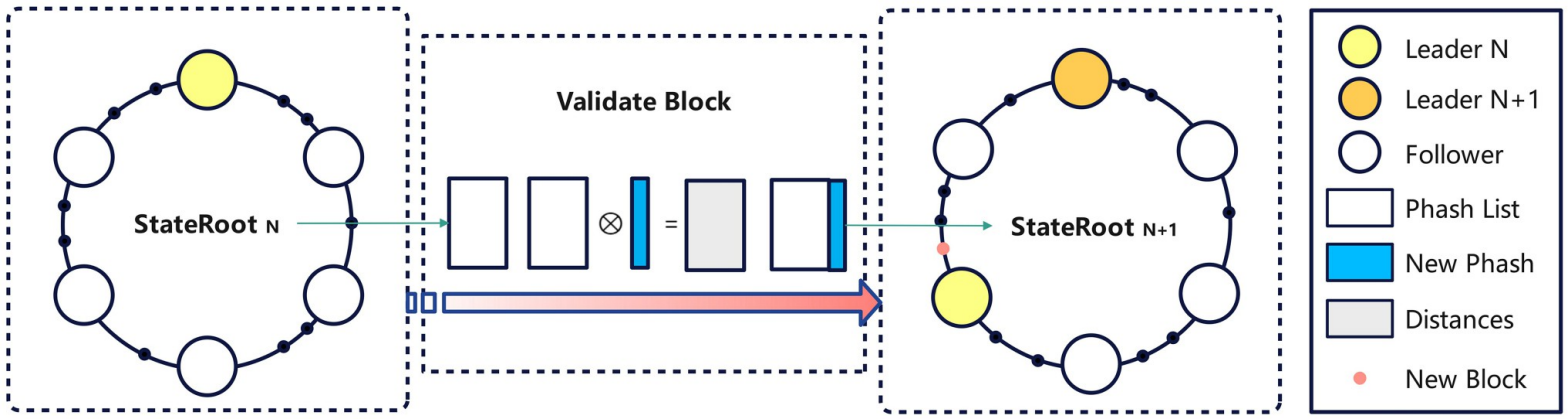
Leader selection process.

The Resolver Election Mechanism refers to the Kademlia protocol-based leader election mechanism for image file cryptographic hashes. It places the network node ID and file hash value in the same domain. The difference is that the distributed hash table based on the Resolver Election Mechanism does not store image data and only stores confirmation tensor pool on each node for large-scale fast retrieval of images. When there is a similar distance from the input image data in the perceptual hash list, it proves that the image may infringe on another’s copyright and is not registered during the registration phase(step 1–3 in Fig 2); instead, it serves as evidence of infringement during the verification phase(step 7–8 in Fig 2). The leader election mechanism is shown in Fig 3. Each time image data is registered and verified, and the Content Provider calculates the cryptographic hash of the image based on the Hamming distance to select the nearest node as the leader. In the next round, the leader is generated in the same way. The leader is always placed at the top of the Fig 3, and when switched, it rotates like a wheeled cockpit, hence its name, Resolver Election Mechanism. The advantages of this election mechanism based on file cryptographic hash values lie in two aspects: on the one hand, it avoids the power consumption caused by PoW consensus algorithms during the competition for blocks; on the other hand, due to the one-way nature and collision resistance characteristics of hash functions, the network becomes more decentralized in the leader election process.

### Multi-leader collaboration

In the Raft consensus algorithm, all nodes start as followers at system startup. A timer is created, and if a Leader’s heartbeat is not received within a specified time, the node becomes a candidate and initiates a vote transaction with other members. If more than half of the members vote for the Candidate, it is promoted to be a Leader; if a Leader sends a heartbeat and receives a response with a more extensive term than its own, it degenerates into a follower. The logical clock (term) election process has a term parameter, which is the logical clock, an integer that increments globally.The leader election mechanism in the Raft consensus algorithm aims to achieve disaster tolerance and rely on heartbeats to ensure that a certain node in the system always possesses the functionality and availability of a Leader. When the Leader suspects communication loss with other nodes, it conducts a leader election switch. Raft divides the time into any length of term and identifies each term with a leader. This ensures that only one Leader exists within a term, thus achieving data consistency. This ensures that data processing is always synchronous and serialized. To provide some degree of asynchronous processing capability, this paper proposes Multi-Leader Collaboration, where multiple leaders may exist simultaneously. These leaders’ final order of block announcements is based on the broadcasting of intermediate states. Like other followers, leaders collect candidate blocks broadcasted by other leaders for verification and sort them in memory in order of their states. When other leaders announce that their blocks are consensus-approved at a future point, the data in memory is released. The parameter alpha represents the number of allowed leaders to coexist simultaneously and is set to 2.

**Fig 4:**
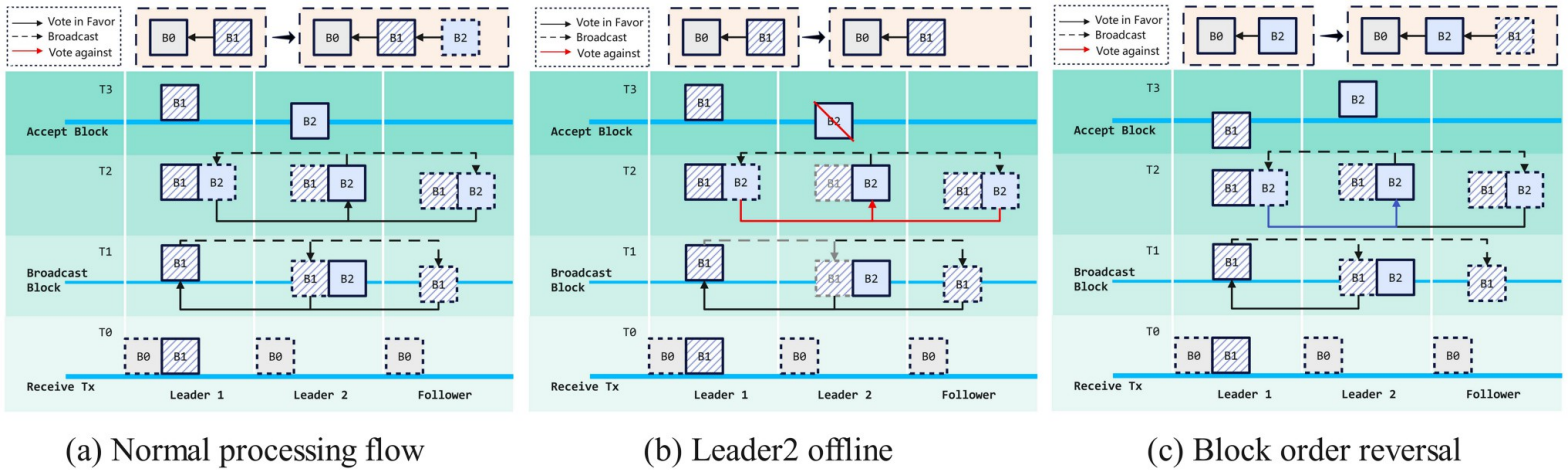
Multi-leader collaboration.

As shown in Fig 4(a), at *T*_0_, Block *B*_0_ has been validated and voted, becoming a block on the blockchain. The memory of each node’s candidate blocks about *B*_0_ is released. At this time, Leader_1_ receives an image registration transaction, and Leader_2_ still needs to become the leader node. When Leader1 runs to the Broadcast Block stage, Leader_2_ is elected in the system, and Leader_2_ will sort the transactions, placing Leader_1_’s candidate block before its local candidate block. Similarly, after Leader_2_ runs to the Broadcast Block stage, it broadcasts its local candidate block, and other leaders will also sort all candidate blocks. K-Raft is a three-phase protocol that starts with each node communication as a phase. The three communications are CALL_BUILD_BLOCK,CALL_VALIDATE_BLOCK, and CALL_STORE.The following is a detailed explanation of K-Raft in stages:

- **Receive Tx Phase**: The first phase is the Receive Tx phase. When a node receives a Tx from the Content Provider, it becomes the leader. In this phase, the leader generates an candidate block locally.
- **Broadcast Block Phase**: The leader who generated the candidate block broadcasts it to other nodes (including other leaders and all followers, but not including the Content Provider of the block). During this phase, if a node drops off, it cannot vote for the candidate block; if that node is not yet in the Broadcast Block phase, as shown in Fig 4(b), it cannot sort the local candidate block (generate the correct block hash). The leader will be rejected during the Broadcast Block because of voting failure.Usually, after this phase, the leader will receive votes from other nodes. If some nodes are owners of certain candidate blocks in front in the order, they will carry their voting situation when voting, as shown in Fig 4(c). If the candidate block sorted later comes out earlier, the order of the blocks will be reversed based on the original order.
- **Accept Block Phase**: The candidate block voted upon through consensus will be uploaded to the blockchain. During this phase, if a node drops off, it cannot obtain a consistent confirmation tensor pool with other nodes. The next time that node votes for another leader’s candidate block, the local confirmation tensor pool will be forced to update.

To incentivize participation in the consensus algorithm, this study has developed a comprehensive set of mechanisms:

1. Image Processing Fee: A fee is defined for users to pay for image processing. This fee is determined by the original image size *RawSize* and a system state parameter *γ*, which reflects the current system load. The formula for calculating the fee is
  $$\begin{array}{c} Cost_{IMG}=RawSize\times \gamma \end{array}$$ (16)
2. Transaction Fee: A proportional transaction fee is charged for each transaction, with the proceeds allocated to the nodes that successfully validate and package the transactions into blocks. The formula for the transaction fee is
  $$\begin{array}{c} Cost_{TX}=Cost_{IMG}\times \left(hops+1\right) \end{array}$$ (17)
  where *hops* denotes the routing hop count for the content provider *CP* to locate the image data leader *Leader*.
3. Consensus Algorithm Engagement: A set of engagement metrics is established based on the different stages of the consensus algorithm, with rewards distributed accordingly. This includes:
  - **Voting Rewards for Followers**: Nodes participating in voting and supporting the final block candidate receive rewards proportional to their voting contribution. The reward formula is:
    $$\begin{array}{c} Cost_{VOTE}=Cost_{IMG}\times e^{-x},x\in \left[1,\frac{2}{3}\times \text{Count}\left(cluster\right)\right] \end{array}$$ (18)
  - **Block Creation Rewards for Leaders**: Nodes that successfully create blocks are awarded a fixed amount of rewards, determined by the difficulty of making the block. The reward formula is:
    $$\begin{array}{c} Cost_{MNIT}=Cost_{TX}-Cost_{VOTE} \end{array}$$ (19)
4. Node Reputation and Penalties: This includes:
  - **Abnormal Handling Rewards**: Nodes that successfully handle anomalies within the consensus algorithm, such as leader transitions or block reordering, receive additional rewards and share the block creation rewards with the block creator.
  - **Penalty Mechanism**: Nodes engaging in cheating or malicious behavior have their rewards deducted and are restricted from participating in the consensus algorithm.

These mechanisms are designed to encourage active participation in the consensus algorithm, thereby enhancing the efficiency and security of the digital image copyright protection system. The reward allocation is based on the nodes’ performance in the consensus algorithm to ensure fair incentivization.

In conclusion, an analysis of the total operational cost of the system is provided. This study begins by thoroughly examining the transaction costs associated with the system. These costs are categorized into three components: uploading image data onto the blockchain, inter-node communication expenses, and storage fees.

The cost of uploading image data onto the blockchain refers to the fees incurred when packaging raw image data into blockchain transactions. This cost is relatively low according to the current fee standards of mainstream blockchain platforms. Inter-node communication costs are the network expenses generated by the extensive communication between nodes during the consensus process. As nodes are deployed in private clouds or local area networks, this portion of the cost is also relatively low. Storage costs are the fees for storing watermarked images on the IPFS distributed storage system. Due to the small size of the watermarked images, this cost is similarly low.

Overall, the system’s transaction costs are mainly derived from the fees associated with uploading data onto the blockchain. This cost may increase for users with high transaction volumes, such as professional photographers. However, this transaction cost is considered acceptable when compared to the benefits of copyright protection offered by blockchain technology. Users can reduce costs by pre-paying fees or adopting batch transactions, among other strategies.

As blockchain technology matures and fee standards evolve, it is anticipated that the system’s transaction costs will gradually decrease. In summary, while the system offers robust copyright protection, the transaction costs remain manageable. In future work, the research will continue to refine the system to reduce transaction costs further, making it more suitable for widespread adoption.

## Experimental setup and performance evaluation

There needs to be more literature on the originality judgment of digital images based on image similarity in blockchain digital image copyright protection. This article continues and expands the ideas of Meng et al. [27]. However, this study does not explain the effectiveness of the perceptual hashing algorithm and watermarking scheme when digital images are attacked. Due to the characteristics of digital images, the scheme’s robustness against attacks is a crucial factor. Therefore, this study provides a more detailed and formal explanation and conducts common 14 attack tests on the scheme proposed by Meng et al. [27]. It also refers to Efficient Cropping-Resistant Robust Image Hashing proposed by Steinebach et al. [36] to make up for the shortcomings of the original scheme. Of course, there are still more robust perceptual hashing methods that can perform better in a broader range of attack scenarios in the future. This is not only the current research deficiency but also one of the directions for our future research.

### Dataset overview

ImageNet is an image database organized based on the WordNet hierarchy (currently limited to only nouns), where hundreds and thousands of images represent each node. The subset of the ImageNet dataset, known as the ImageNet Large Scale Visual Recognition Challenge (ILSVRC), has become the most popular subset of the dataset. It consists of 1000 object classes used to benchmark image classification algorithms. For this experiment, we have used the first 100 classes of ILSVRC and randomly selected 40 images per category as ImageNet-100 dataset, resulting in a total of 4000 images for the experimental dataset.

### Verifying the feasibility of perceptual hash and Hamming distances

This study computes the perceptual hashes to ascertain the utility of perceptual hashing combined with Hamming distance for digital image copyright protection. We analyzed the Hamming distances for images across various categories within the ImageNet-100 dataset. The experimental findings reveal that the Hamming distances between different images within the same category are substantial, indicating that the perceptual hashing algorithm can effectively distinguish between distinct images within a single category.This demonstrates the feasibility of employing such an approach for copyright protection purposes.

**Fig 5:**
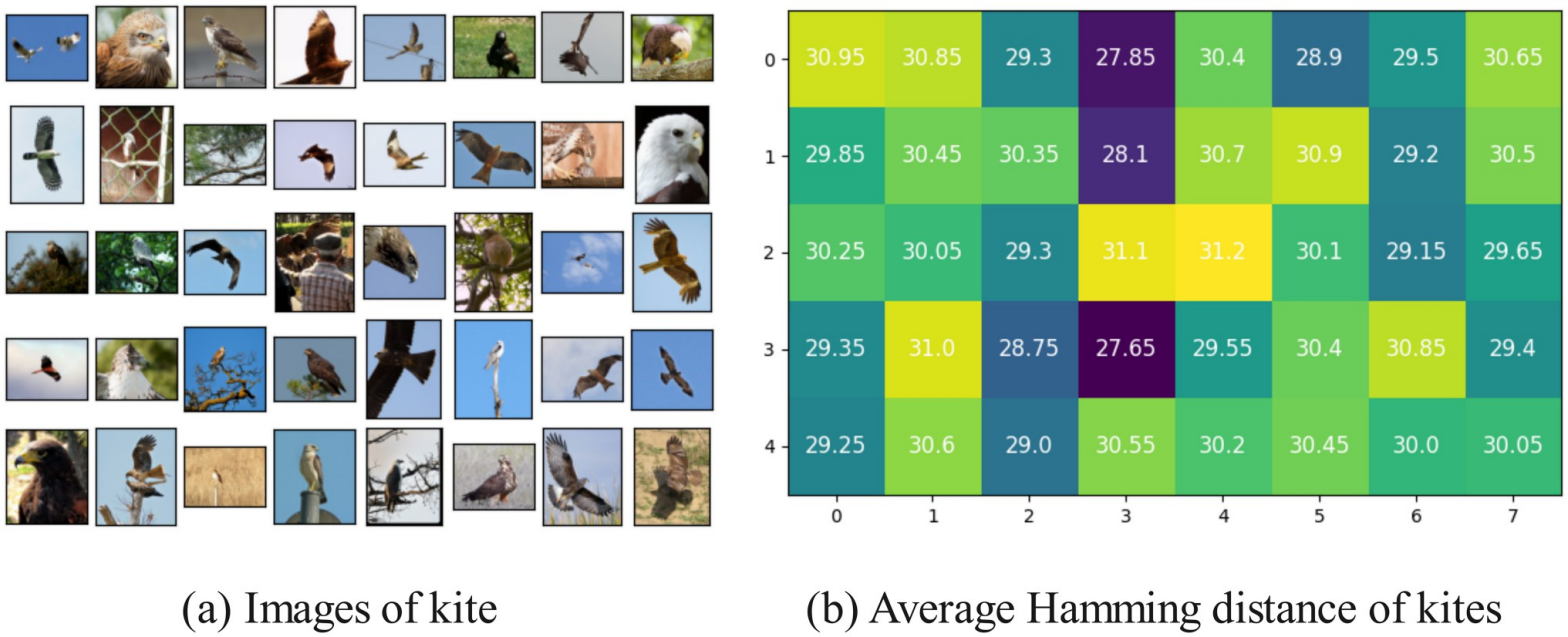
Average Hamming distance of perceptual hashes of images of the same category.

First, the PHA and average Hamming distance between any image in the 100 classes and other images within the same class were calculated. Fig 5(a) shows images of the kite under the n01608432 folder in the ImageNet-100 dataset, and Fig 5(b) shows the average Hamming distance between any image in the kite category and other images within the same category. It can be seen that the minimum value is 27.65, indicating a large Hamming distance between different images.

### Verifying the impact of attacks on watermark extraction

The robustness of watermark extraction is a critical metric for evaluating digital image copyright protection systems. This study subjected the same image to 14 common attacks and assessed the efficacy of watermark extraction. The experimental results demonstrate that the perceptual hashing algorithm is resilient to scaling, noise, brightness, and color variations, yet it is susceptible to cropping attacks. To address this vulnerability, we employed a robust image hashing algorithm with enhanced resistance to cropping, which consistently exhibited superior performance against all 14 attack types.

**Fig 6:**
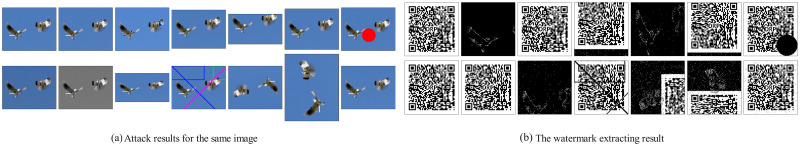
The extraction of watermarks under different attacks.

**Fig 7:**
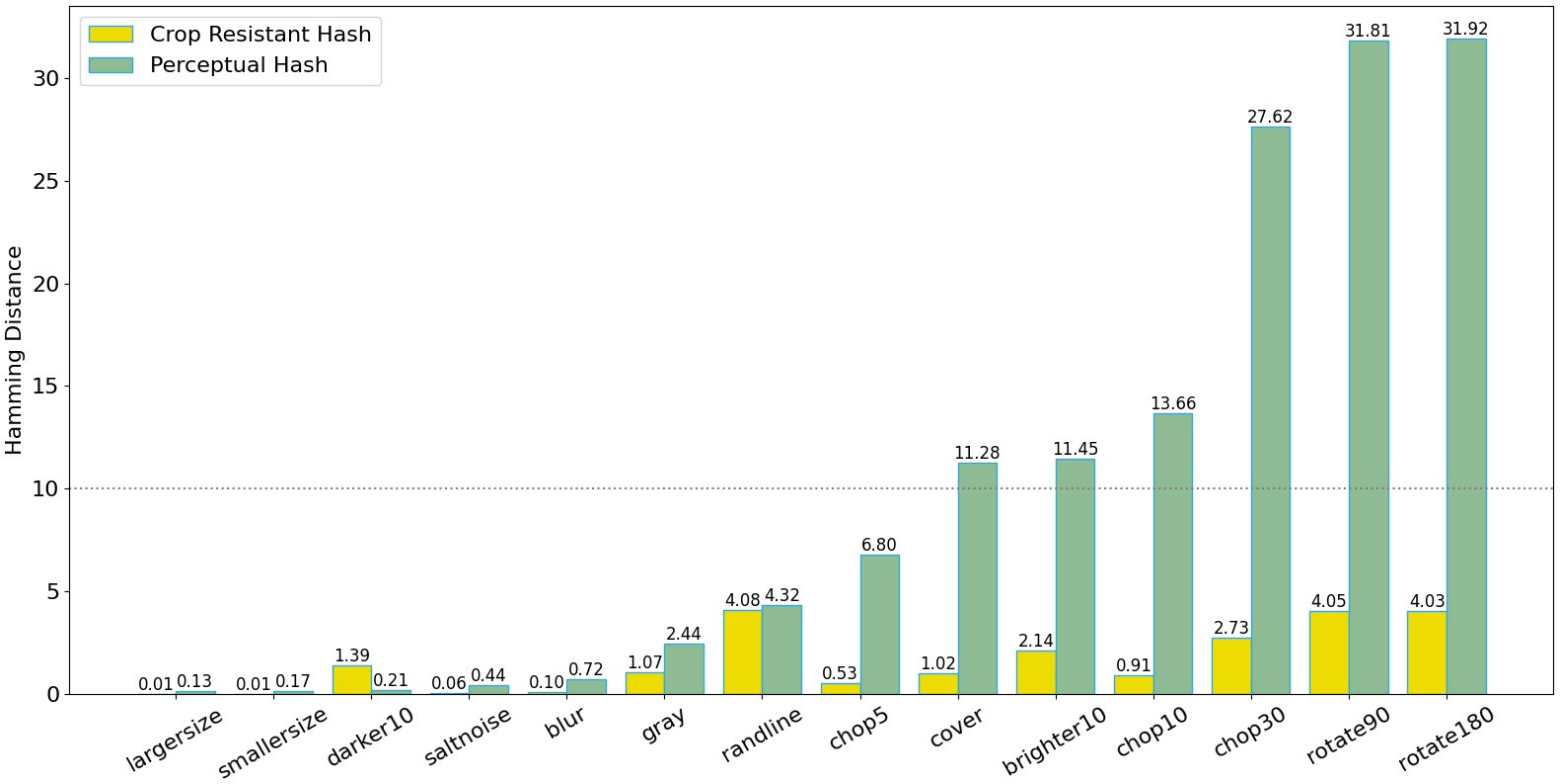
The average Hamming distance under different attacks.

Fig 6(a) shows that the experiment applied 14 common attacks to the same image, such as flipping, cropping, noise, brightness, and color change. The employed PHA approach is a 64-bit scheme, and the Hamming distance threshold was set at less than 10, indicating that images had a similarity higher than 85%. Under this condition, as shown in Fig 7, the PHA strongly resists scaling, noise, brightness, and color change attacks.Because the PHA algorithm is not resistant to cropping attacks, we adopted the more robust efficient cropping-resistant robust image hashing algorithm. It can be seen that this algorithm has good defense effects against 14 common attacks.

Still, solutions to this problem are relatively simple and require reversing the image before calculating the perceptual hash. Thus, the perceptual hash method has strong robustness when the image data is not extensively masked or cropped. This experiment verifies the reasonableness of using perceptual hashes and Hamming distances to analyze image similarity.

**Fig 8:**
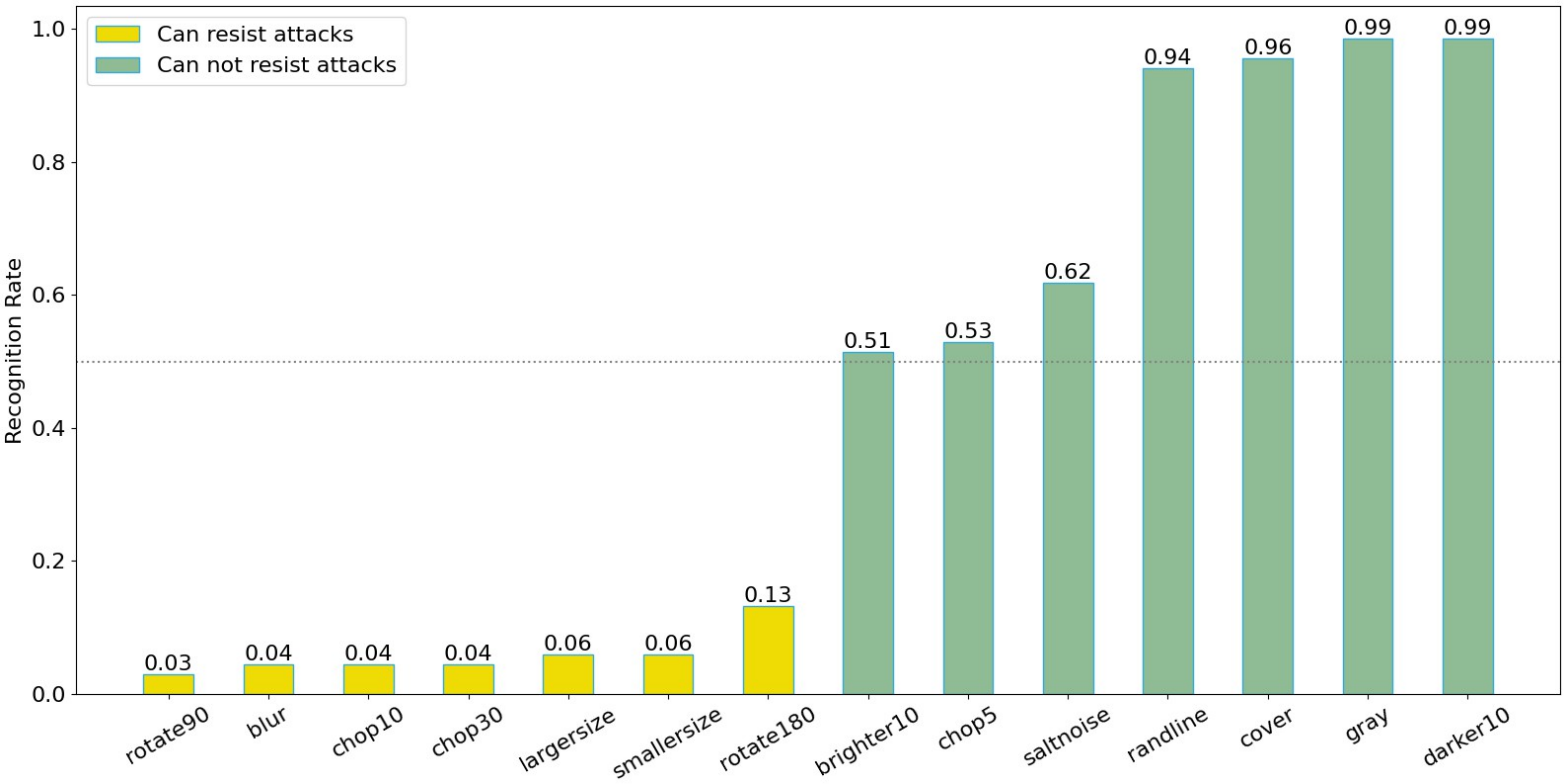
The watermark extracting ability under different attacks.

The watermark extraction ability refers to successfully extracting block data from the QR-code watermark after being attacked. When the embedded watermark image is attacked, the corresponding watermark in the image will also be destroyed. Therefore, the extraction ability of watermarks after being attacked is also a key indicator. As shown in Fig 6, the same image has been demonstrated under 14 types of attacks, and Fig 6(b) shows the extraction of watermarks under 14 types of attacks. The Fig 8 indicates that for each randomly selected image from each of the 100 categories, the extraction ability of watermarks under different attacks is shown as follows. In this paper, 50% is set as having the ability to resist specific attacks. It can be seen that watermarks cannot be extracted after cropping, scaling, rotating, and blurring. In summary, the attack resistance capabilities of Perception Hashing, Watermarking Technology, and Robust Perception Hashing are shown in the Table 5.

**Table 5:** Resilience of scheme components to attacks.

Attack Type	PHA 64bits	Watermarking	Crop Resistant Hash
largersize	√	×	√
smallersize	√	×	√
darker10	√	√	√
saltnoise	√	√	√
blur	√	×	√
gray	√	√	√
randline	√	√	√
chop5	√	√	√
cover	×	√	√
brighter10	×	√	√
chop10	×	×	√
chop30	×	×	√
rotate90	×	×	√
rotate180	×	×	√

### Efficiency test of consensus algorithm

This study benchmarks the performance of the DRPChain consensus segment implemented with the K-Raft single-leader, K-Raft dual-leader coordination, and the native Raft consensus algorithms. The findings indicate that, with a node count of up to eleven, the dual-leader coordination exhibits performance comparable to or slightly superior to the single-leader model. The native Raft consensus algorithm, ill-suited for leader handovers within single transactions, requires leader elections after each block’s publication, incurring additional time overhead. Experimental outcomes corroborate that the K-Raft consensus algorithm aligns more with the proposed digital image copyright protection scheme than the native Raft protocol.

**Fig 9:**
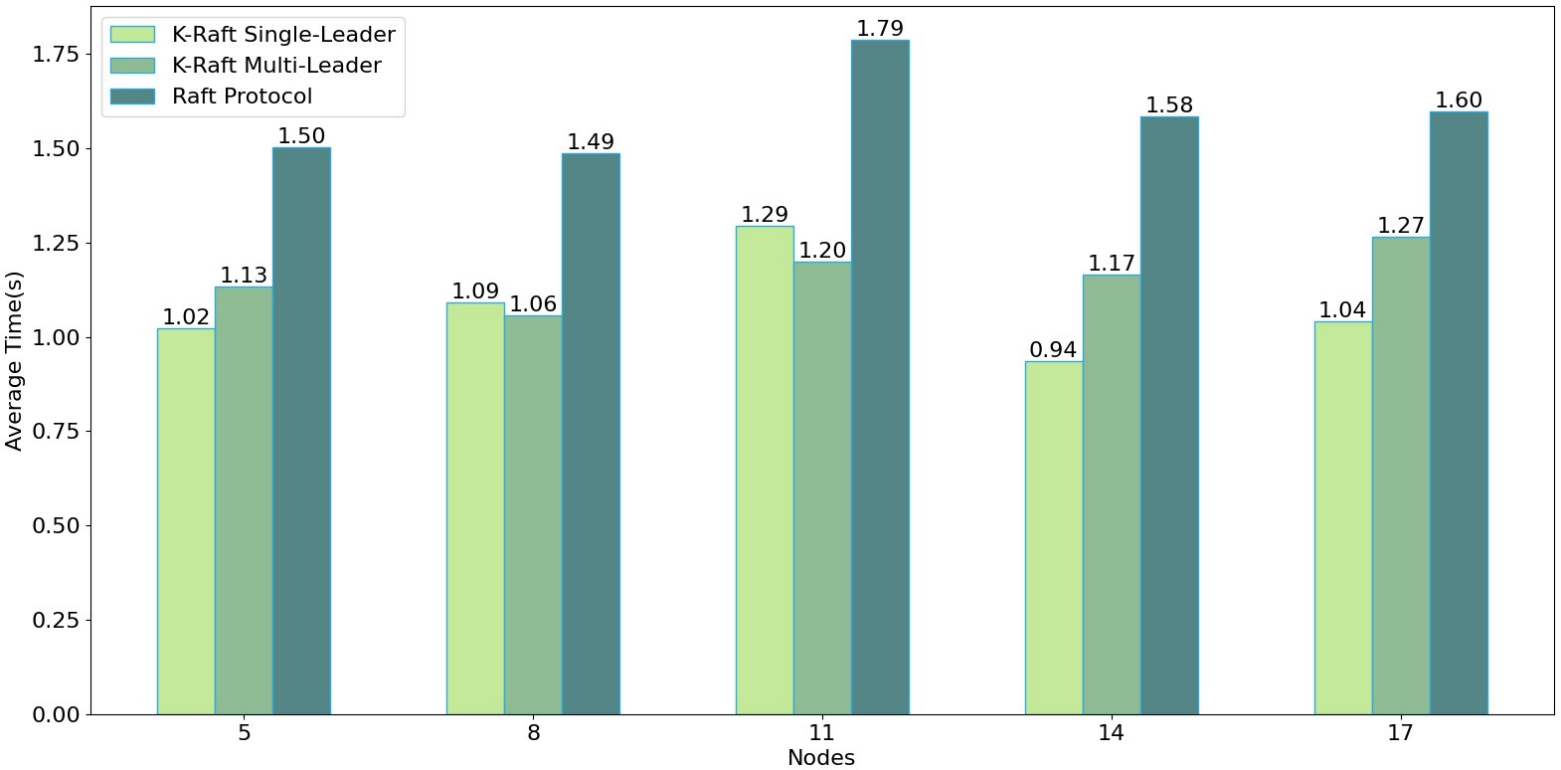
Efficiency of different consensus algorithms.

Based on virtualization technology, the experimental node hardware environment is configured with an Intel Core (TM) i7-10700@2.90Ghz CPU, 16GB memory, and three nodes per machine. The software is implemented using Python 3.7. Each node sends transactions to the leader of the transaction every two seconds (with a random upward fluctuation not exceeding 1 second) and stops sending after sending 1000 transactions. The DRPChain consensus part implemented by the K-Raft single leader, K-Raft dual leader coordination, and native Raft consensus algorithm were analyzed, respectively. As shown in the Fig 9, due to the inevitable need for occasional sorting and reversing of candidate blocks in dual leader coordination, the time complexity of a single transaction execution block is increased. Under no more than eleven nodes, the performance is comparable to that of a K-Raft single leader and even slightly higher than that of a single leader under eight and eleven nodes. Since the native Raft consensus algorithm is unsuitable for leader switching in a single transaction, the leader must run for election after each block is released, which consumes some time. This shows that compared to the native Raft protocol, the K-Raft consensus algorithm is more usable for the proposed image data copyright protection scheme.

### Anomaly analysis of block generation

This study thoroughly analyzes anomalies encountered during the generation of proposed blocks. Findings indicate that the probability of watermark extraction failure is negligible. In contrast, the likelihood of leader legitimacy verification failure increases progressively with the number of nodes, predominantly due to network instability that hampers the rapid dissemination of log information to all nodes. In general, adopting a dual-leader collaboration scheme markedly enhances system efficiency. Specifically, when the number of nodes does not exceed eleven, the proposed scheme outperforms the native Raft protocol regarding transaction processing time and block generation error rates, making it more suitable for the proposed system requirements.

**Fig 10:**
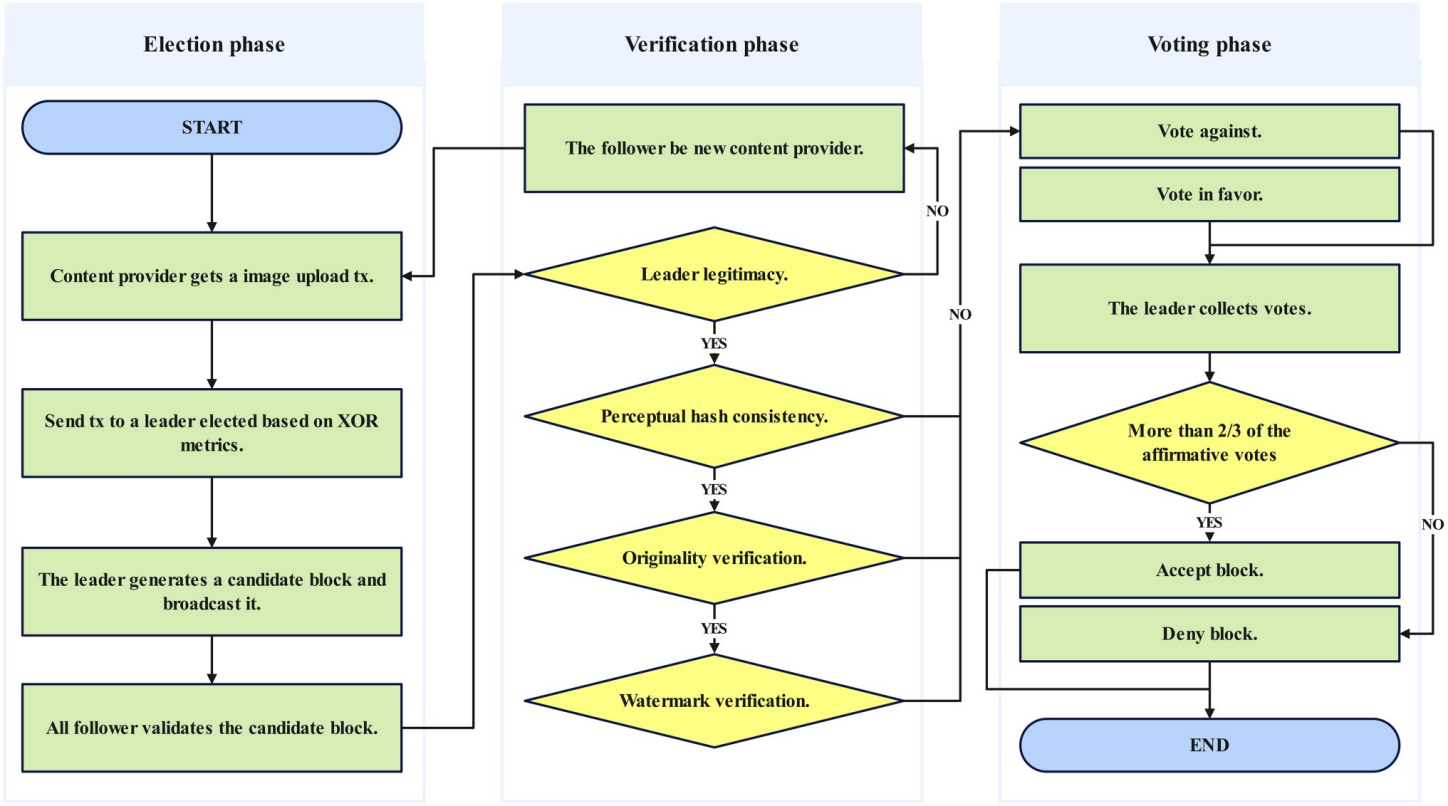
Consensus process.

**Fig 11:**
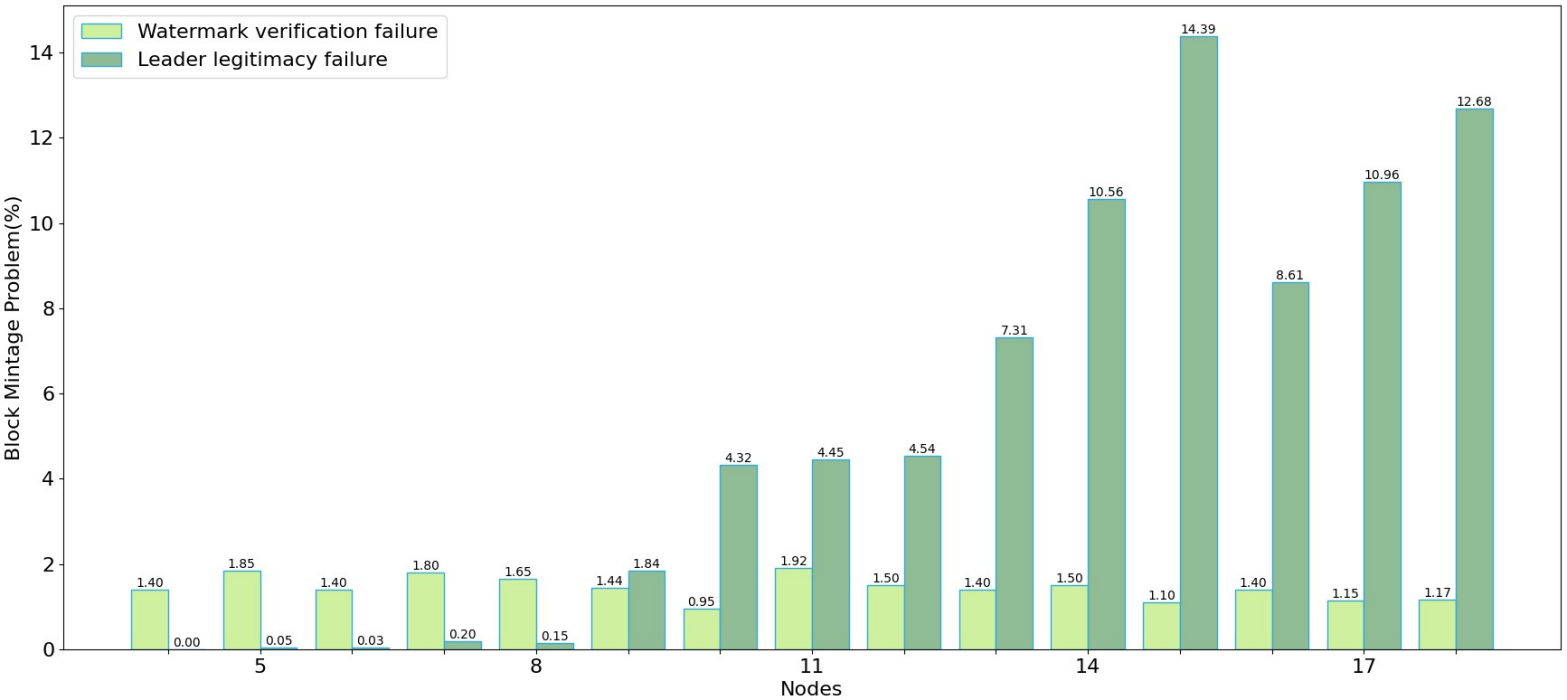
Block mint failure distribution.

As shown in Fig 10, four steps are involved in generating a block on the candidate block. Data related to all anomalies in block generation were recorded from nodes 5–17. Two issues caused anomalies during the experiment:

**Watermark extraction failure**. Watermark extraction failure refers to the inability to extract valid QR-code block information from images with embedded watermarks. The probability of this situation is minimal. As shown in the Fig 11, it remains consistently around 1%.

**Leader legitimacy failure**. There were two situations where the follower calculated the leader did not match the sender of the candidate block or the length of the local confirmation tensor pool entry of the follower was greater than that of the leader’s confirmation tensor pool entry. Since the leaders were selected normally in this experiment, these anomalies were caused by the leader node’s log not being updated in time. As shown in the figure, with the increase in node quantity, the error rate in block generation continues to rise, and blocks cannot be correctly verified due to the leader node not receiving the latest logs in time.

As shown in the Fig 11, it can be observed that as the number of nodes increases, due to the instability of the network, logs cannot be quickly propagated to all nodes, increasing the probability of Leader legitimacy failure. The node numbers are divided into 5–17, and each class of node numbers has a system with a single leader, a dual leader, and the native Raft protocol. The native Raft protocol fails to switch leaders quickly, leading to increased data inconsistency and Leader legitimacy failure. The dual leader situation shows no significant difference from the single leader situation within 11 node numbers. However, as the number of nodes increases, the probability of Leader legitimacy failure gradually increases.

In blockchain-based digital image copyright protection systems, selecting consensus algorithms is crucial for system performance and security.Common consensus algorithms include PoW [30, 31], PBFT [28], Raft, and the proposed K-Raft algorithm. PoW relies on computational power competition for block packaging rights, offering high security but substantial resource consumption and potential for centralization. PBFT, a practical Byzantine fault-tolerant algorithm, provides high consensus efficiency but low decentralization. Based on log-replication state machines, Raft offers high consensus efficiency but lacks leader node switching speed.Addressing this, the K-Raft algorithm introduces Kademlia routing tables and hash-distance-based leader node selection, achieving more decentralized leader selection. It also allows for multiple temporary leaders, enhancing system efficiency. Experiments show that K-Raft outperforms PoW, PBFT, and Raft in decentralization, leader selection, and system efficiency, making it more suitable for digital image copyright protection.

**Fig 12:**
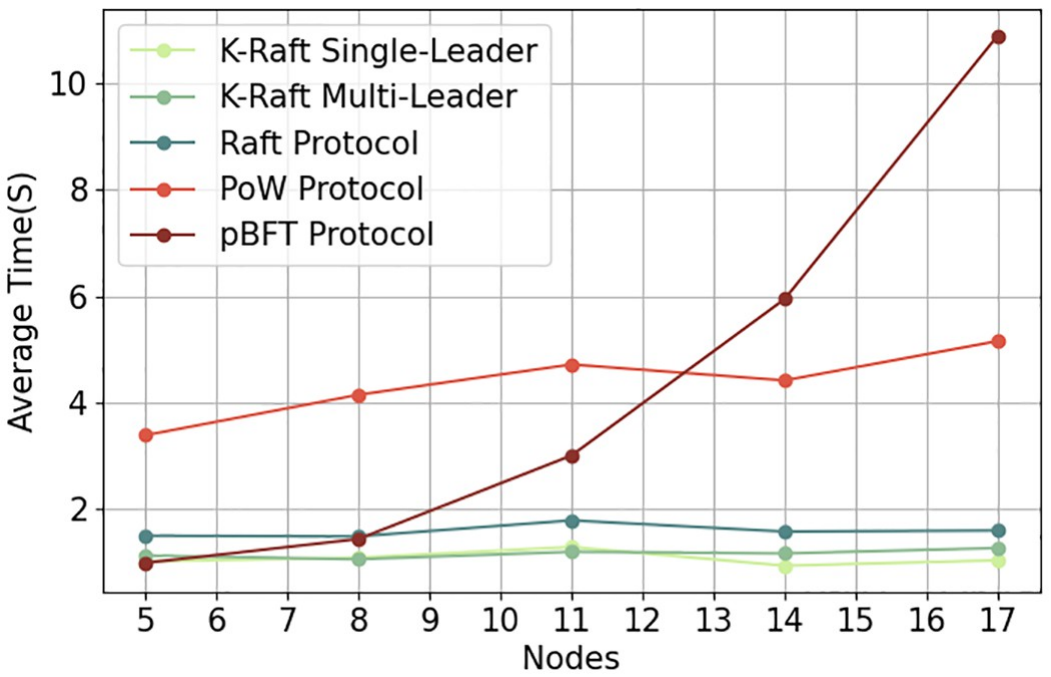
Comparison of schemes.

As shown in Table 6 and Fig 12, analyzing the efficiency of five consensus algorithms (K-Raft-S et al.) involved detailed comparative studies based on experimental data.

**Table 6:** Comparison of schemes.

	5	8	11	14	17
K-Raft S	1.02	1.09	1.29	0.94	1.04
K-Raft M	1.13	1.06	1.20	1.17	1.27
Raft	1.50	1.49	1.79	1.58	1.60
PoW [30, 31]	3.39	4.51	4.72	4.42	5.16
pBFT [28]	0.99	1.44	3.01	5.95	10.9

Initially, PBFT exhibited high efficiency, with values of 0.99, 1.44, 3.01, 5.95, and 10.90. However, PBFT’s efficiency plummeted as tests progressed, performing worst among all algorithms.This suggests that while PBFT excels in austere or low-load environments, its performance significantly deteriorates under complex or high-load conditions. K-Raft-S and K-Raft-M showed relative stability throughout testing, with minimal efficiency fluctuations: K-Raft-S: [1.02, 1.09, 1.29, 0.94, 1.04], K-Raft-M: [1.13, 1.06, 1.20, 1.17, 1.27]. K-Raft-M generally outperformed K-Raft-S, attributed to its multi-leader collaboration mechanism.

Raft’s efficiency was between the K-Raft series and PoW, showing robust performance without significant fluctuations: 1.50, 1.49, 1.79, 1.58, and 1.60. This indicates Raft’s ability to handle tasks of varying complexity while maintaining high efficiency. PoW’s efficiency was consistently low across all tests, with values of 3.39, 4.15, 4.72, 4.42, and 5.16, decreasing with test complexity.This aligns with PoW’s computation-intensive nature, which sacrifices efficiency for high security.

In conclusion, the efficiency differences among consensus algorithms stem from their design principles and targeted application scenarios. The choice of consensus algorithm should be based on specific system requirements for security and efficiency. For instance, PBFT and PoW may be more suitable for scenarios demanding high security and fault tolerance, while K-Raft series and Raft might be preferable for efficiency-driven applications.

K-Raft enhances Raft with a multi-leader collaboration mechanism, achieving soft leader switching. Compared to native Raft, K-Raft introduces the ReSolver election mechanism, using image data hash values for leader selection, ensuring fairness and decentralization. It also supports the coexistence of multiple leaders, improving system efficiency and throughput and reducing block error rates. Experimental results show that K-Raft improves efficiency by approximately 300ms and reduces block error rates by about 2% compared to native Raft.Designed specifically for image copyright protection, K-Raft’s consensus algorithm is better suited for this application scenario, demonstrating advantages in decentralization, election fairness, system efficiency, and throughput.

## Discussion

This section will delineate additional aspects not emphasized in the main text, intertwining experimental and evaluation results.

During the deployment of blockchain nodes for the digital image copyright management system, selecting appropriate hardware configurations is contingent upon the node’s role. The configurations are distinct based on the node’s role, with full nodes storing comprehensive data and executing the full consensus algorithm. In contrast, light nodes store minimal data and perform a lightweight consensus algorithm. Specifically, full nodes require high-performance CPUs and memory to assume roles such as *Follower*, *Leader*, or content provider *CP*. In contrast, light nodes are configured with less sophisticated components, restricted to the *Follower* role in the consensus mechanism.

Regarding communication security, the system employs SSL/TLS encryption to safeguard inter-node communications and implements access control mechanisms. Experimental tests have assessed the system’s performance under varying node counts and transaction volumes. The results indicate the system exhibits good scalability with up to 11 nodes. To accommodate larger transaction volumes, further optimization of the system architecture will be pursued, including the introduction of lightweight nodes and an increase in the number of nodes, to ensure system performance in a large-scale environment. The total number of full nodes is maintained within 5 to 11 to preserve high consensus efficiency. In contrast, the abundance of lightweight nodes enhances the efficiency of the voting process. Additionally,real-time monitoring of all nodes is conducted to ensure system stability.Finally, the network topology is designed to align with business requirements, enhancing system availability.

To enhance usability, the user interface should be streamlined to offer an intuitive and straightforward operational interface with reduced steps. For developers, developing user-friendly application programming interfaces (APIs) that encapsulate the underlying blockchain technical intricacies is essential for seamless integration into applications. A one-click deployment option for blockchain nodes is provided for non-technical users, simplifying the deployment process and supporting multiple access methods, including web interfaces and mobile applications, to cater to diverse user requirements. Economic incentive policies can also enhance the engagement of non-technical users. These measures aim to reduce system complexity, enhance usability, and facilitate broader user adoption.

In response to the issue of blockchain technology dependency, this study, within the context of current blockchain technology advancements, acknowledges that while leveraging blockchain as the underlying architecture enables decentralized trust, it inherently entails efficiency trade-offs. To mitigate these challenges, this research presents a suite of enhancements: evaluating the scalability of blockchain platforms and selecting or designing scalable blockchain architectures to enhance transaction processing capabilities; analyzing the business impact under various fault scenarios and formulating continuity plans; and conducting regular technical risk assessments to devise countermeasures. These measures collectively aim to reduce the system’s reliance on blockchain technology, enhancing its stability and security.

To address the interoperability challenges of the proposed system with other blockchain platforms and existing digital ecosystems, this study initially assesses the compatibility and interoperability of the system with mainstream copyright management systems, focusing on smart contract interfaces, data exchange formats, and process integration, and considering the provision of currency exchange services between platforms such as Bitcoin and Ethereum. Subsequently, it proposes measures for improvement, including optimizing interface design, unifying data formats, and streamlining process integration to enhance system interoperability. Specific strategies include defining uniform data exchange interfaces, employing standardized data formats, simplifying cross-system operation processes, researching and integrating cross-chain technologies, supporting multiple blockchain platforms, and offering currency exchange services between blockchain platforms. Through these initiatives, the study aims to bolster the interoperability of the proposed system with other blockchain platforms and existing digital ecosystems, fostering widespread adoption and synergistic effects.

From the outset of this study, a focus was placed on optimizing the scalability of the system design. A layered architecture was initially adopted, separating data processing from business logic. The data layer harnesses IPFS distributed storage technology to address the challenges of large-scale data storage, while the business layer, built on blockchain, stores only critical metadata, significantly alleviating storage and computational burdens.

Furthermore, this study innovatively optimized the consensus algorithm by developing the K-Raft algorithm. By introducing a multi-leader collaboration mechanism, this algorithm enables multiple leader nodes to process transactions in parallel, markedly enhancing the system’s throughput. Additionally, a file-hash-based leader election mechanism is employed, circumventing the issue of resource wastage prevalent in traditional consensus algorithms.

To validate the system’s scalability, performance evaluations were conducted in test environments with varying numbers of nodes. Experimental results demonstrate that as the number of nodes increases, the system’s transaction throughput and latency remain stable, affirming the efficacy of the system design.

## Conclusions

This paper introduces DRPChain, a novel digital image copyright management system that leverages blockchain technology. It contributes significantly by presenting a comprehensive analysis and enhanced solutions, such as adopting an efficient cropping-resistant robust image hashing algorithm to resist common image attacks. Experimental results demonstrate that the proposed algorithm performs better against 14 common image attacks, with an average success rate of 85% in watermark extraction, which is 10% higher than the original scheme.

Additionally, the paper designs the K-Raft consensus algorithm tailored for image copyright protection based on the Kademlia protocol and the Raft consensus algorithm. Comparative experiments with the native Raft algorithm show that K-Raft reduces the block generation error rate by 2% and improves efficiency by approximately 300ms, making it more suitable for digital image copyright protection.

Furthermore, benchmarking against PoW and PBFT consensus algorithms reveals K-Raft’s efficiency, decentralization, and system throughput superiority. In tests, K-Raft demonstrates an average efficiency of 1.2× that of Raft, significantly higher than PoW’s average efficiency of 4.6× and PBFT’s average of 3.0×. This highlights K-Raft’s ability to maintain high efficiency even as the system scales. Additionally, K-Raft achieves a more decentralized leader selection mechanism than PoW and PBFT, with a hash-based leader election process that ensures fairness and decentralization. These advantages make K-Raft a more suitable choice for digital image copyright protection.

In future work, the aim is to extend the protection to various digital media types, including video, text, and music. By combining perceptual hashing and deep learning techniques, multi-modal feature extraction and matching can be utilized to achieve cross-modal copyright protection. This will necessitate further optimization of the consensus algorithm to meet broader application needs, focusing on improving efficiency. Overall, this research contributes to digital copyright protection using blockchain technology, laying a solid foundation for future exploration.

## Supporting information

S1 Appendix All the attack methods employed in this study. The table describes all the specific characteristics of all the attack methods employed in this study.
(ZIP)
