# Supplementary material for: DRPChain: A new blockchain-based trusted DRM scheme for image content protection
Source: PLoS One. 2024 Sep 19;19(9):e0309743. doi: 10.1371/journal.pone.0309743 (PMC11412486; doi:10.1371/journal.pone.0309743)
Supplement: S1 Appendix — The table describes all the specific characteristics of all the attack methods employed in this study. (ZIP) [file pone.0309743.s001.zip › S1_Appendix.pdf]

Appendix

Table 1. All the attack methods employed in this study.

Attack Type	Detail
Scaling Attack	Alters the image size by enlarging or reducing it,aiming to disrupt the embedded watermark
Brightness Adjustment Attack	Modifies the image's brightness levels, affecting its luminance and hindering watermark extraction
Rotation Attack	Rotates the image to change its orientation, thereby confusing the watermark recognition process.
Noise Addition Attack	Introduces random noise into the image to interfere with the accurate extraction of the watermark.
Blurring Attack	Applies blurring techniques to the image, reducing its quality and impeding watermark extraction.
Grayscale Conversion Attack	Converts color images to grayscale, decreasing color information and affecting watermark identification.
Random Line Insertion Attack	Adds random lines to the image, disrupting the precise extraction of the watermark.
Cropping Attack	Removes parts of the image through cropping, attempting to damage the embedded watermark.
Overlay Attack	Adds a layer over the image to conceal or damage the watermark.
Color Transformation Attack	Alters the image's color scheme, aiming to obfuscate the watermark's recognition.
Brightness Enhancement Attack	Increases the image's brightness, impacting the visibility of the watermark.
30% Cropping Attack	Crops 30% of the image area to test the watermark's resistance to cropping.
90° Rotation Attack	Rotates the image by 90 degrees to change its orientation , testing the watermark's resistance to rotation.
180° Rotation Attack	Rotates the image by 180 degrees to change its orientation, testing the watermark's resistance to rotation.

The Table 1 describes all the specific characteristics of all the attack methods employed in this study.
